# Age effects on explicit and implicit memory

**DOI:** 10.3389/fpsyg.2013.00639

**Published:** 2013-09-23

**Authors:** Emma V. Ward, Christopher J. Berry, David R. Shanks

**Affiliations:** ^1^Psychology Department, Middlesex UniversityLondon, UK; ^2^School of Psychology, Plymouth UniversityPlymouth, UK; ^3^Division of Psychology and Language Sciences, University College LondonLondon, UK

**Keywords:** aging, implicit memory, priming, recognition, models of memory

## Abstract

It is well-documented that explicit memory (e.g., recognition) declines with age. In contrast, many argue that implicit memory (e.g., priming) is preserved in healthy aging. For example, priming on tasks such as perceptual identification is often not statistically different in groups of young and older adults. Such observations are commonly taken as evidence for distinct explicit and implicit learning/memory systems. In this article we discuss several lines of evidence that challenge this view. We describe how patterns of differential age-related decline may arise from differences in the ways in which the two forms of memory are commonly measured, and review recent research suggesting that under improved measurement methods, implicit memory is not age-invariant. Formal computational models are of considerable utility in revealing the nature of underlying systems. We report the results of applying single and multiple-systems models to data on age effects in implicit and explicit memory. Model comparison clearly favors the single-system view. Implications for the memory systems debate are discussed.

With the age distribution of the global population steadily increasing, there is great importance in studying and understanding changes in learning and memory in later adulthood. As well as providing a clearer picture of how these functions are affected by cognitive decline, research in this area can shed considerable light on the underlying structure of memory. This has substantial and wide-reaching implications. It is now well-established that explicit memory, the conscious experience of remembering previously learned information, declines with age and moreover the rate of decline is an important predictor of dementia (Wilson et al., 2011). In contrast, there have been numerous reports over the last few decades of age-invariant implicit memory in healthy subjects. Implicit memory, it is often argued, occurs outside of awareness, and is evident when prior experiences affect (e.g., facilitate) performance on tasks that do not require conscious recollection of those experiences (Schacter, 1987). Reports of differential effects of age on explicit and implicit memory have led many to conclude that the two are driven by functionally distinct memory systems. If true, then preservation of implicit memory might open up significant opportunities to remediate cognitive decline, aiding such important real-life demands as acquiring face-name pairs or learning medication routines. Moreover, because it has been suggested that implicit memory is preserved in healthy older individuals but not those with early Alzheimer's Disease, well-designed implicit tasks might provide a useful diagnostic tool for pathological aging (see Fleischman, 2007). In this article we review observations that have led researchers to conclude that implicit memory is preserved with age. We discuss various issues that undermine this interpretation, as well as the notion that the two forms of memory are controlled by separate systems.

## Explicit and implicit memory in normal aging

Perhaps the best documented cognitive feature of growing older is that it is associated with a decline in the capacity to consciously learn and remember. Healthy older individuals often experience difficulties in remembering recently learned information, and typically perform worse than their younger counterparts on laboratory tests of explicit memory (reviewed in Mitchell, 1989; Light, 1991; Kausler, 1994). Such tests instruct participants to deliberately attempt to recall or recognize specific information from a prior episode. For example, in a recognition task, participants study a series of stimuli such as pictures or words and are later asked to discriminate between previously studied (old) and new items.

Explicit memory is thought to decline steadily throughout late adulthood. A study by Fleischman et al. (2004) reported progressive decline in individuals with a mean age of 78.6 years over a 4-year period on a battery of explicit tests involving immediate and delayed recall and recognition of stories, numbers, and words (see also Hultsch et al., 1992; Christensen et al., 1997; Davis et al., 2001). There is also an abundance of evidence from cross-sectional studies that individuals over the age of 60 perform significantly worse than individuals in their twenties on tests of recall and recognition (reviewed in Mitchell, 1989; Light, 1991).

Given the clear decline in explicit memory with age, there has been a profound interest over the past few decades in establishing whether implicit memory also declines. Implicit memory is evident when previously learned information affects (e.g., facilitates) performance on tasks that do not require the individual to consciously recollect the information. Previously encountered stimuli have a processing advantage over stimuli that were not previously encountered, a phenomenon known as repetition priming (henceforth priming). For example, seeing the word “cake” increases the likelihood that it will later be used to solve the word-fragment “c____e” even though there are several possible solutions. This type of word-completion task is commonly used in the laboratory to measure priming (e.g., Park and Shaw, 1992; Small et al., 1995; Spaan and Raaijmakers, 2011), and is usually disguised as an unrelated task. That is, following an initial phase in which a series of words are studied, participants are subsequently asked to complete word-fragments or stems with the first word that comes to mind, and no reference is made to the prior study phase. Perceptual identification is another commonly employed task that is used to compare the performance of different age groups (e.g., Fleischman and Gabrieli, 1998; Buchner and Wippich, 2000; Light et al., 2000; Mitchell and Bruss, 2003). Here, following a seemingly unrelated task in which a series of stimuli (usually pictures or words) are viewed, old and new items are presented either very briefly or in a degraded form, and participants are simply asked to identify them. Priming is revealed in the speeded or more accurate identification of old relative to new items.

The majority of studies that have investigated priming in normal aging have reported that it does not decline significantly (reviewed in Mitchell, 1989; Fleischman and Gabrieli, 1998; Fleischman, 2007). Several longitudinal studies have demonstrated stable priming with advancing age despite substantial declines in explicit memory (Hultsch et al., 1992; Christensen et al., 1997; Davis et al., 2001; Fleischman et al., 2004). Moreover, many cross-sectional studies employing a range of different tasks have reported non-significantly different priming between groups of young and older adults (e.g., Mitchell et al., 1990; Light et al., 1992; Park and Shaw, 1992; Schacter et al., 1992; Mitchell and Bruss, 2003; Wiggs et al., 2006; Soldan et al., 2009; Spaan and Raaijmakers, 2011).

The apparent sparing of implicit memory (priming) with age in the face of a clear decline in explicit memory has been a highly influential observation in the memory systems debate. It has been heavily cited in the literature as providing evidence that the two forms of memory are qualitatively distinct and driven by functionally independent cognitive and neural systems (e.g., Schacter, 1987; Tulving and Schacter, 1990; Schacter and Tulving, 1994; Squire, 1994, 2004, 2009; Gabrieli, 1998, 1999). Supporters of this multiple-systems perspective have argued that the decline in explicit but not implicit memory demonstrates that an explicit system succumbs to natural age-related changes in the brain, while an implicit system does not. There are, however, salient problems with the interpretation that implicit memory is completely spared with age.

## Implicit memory: spared or impaired in normal aging?

The idea that implicit memory remains stable as we age has come to be widely accepted, yet not all studies demonstrate preserved priming in older individuals. There are cases in the literature in which priming in older adults is significantly reduced in comparison to that in young adults (e.g., Chiarello and Hoyer, 1988; Abbenhuis et al., 1990; Davis et al., 1990; Hultsch et al., 1991; Ward et al., 2013). Moreover, in published studies that claim to have revealed preserved priming in older individuals, performance has most often been numerically reduced in these individuals compared to the young (see Fleischman and Gabrieli, 1998). Could there be a genuine decline in priming with age that often goes undetected? If the effect is very small, then because the majority of studies have used relatively small sample sizes, the answer to this question may be yes. Statistical power to detect small but real age effects may have been too low in many studies (a detailed discussion of this issue is provided in Mitchell and Bruss, 2003). La Voie and Light (1994) conducted an extensive meta-analysis, which uncovered a small but significant age effect on priming, calling into question the soundness of drawing a distinction between explicit and implicit memory systems. A decline in both forms of memory with age could point to a general impairment of a unitary memory system.

If both explicit and implicit forms of memory decline with age, why is the effect so much smaller on measures of implicit memory, to the extent that changes are difficult to detect statistically? One issue is that implicit tasks often have lower reliability than explicit tasks (see Buchner and Wippich, 2000; LeBel and Paunonen, 2011; Ward et al., 2013). To illustrate this, consider the comparison that is frequently made between performance on recognition and word-completion tasks. In a recognition task the goal to discriminate old from new items is relatively rigid, whereas the instructions in word-completion tasks—to complete stems or fragments with the first word that comes to mind—allow a considerable amount of flexibility in performance strategy. Put differently, it is clear to the individual that whichever strategy maximizes discrimination accuracy in recognition is better than all other strategies; in word-completion, by contrast, any strategy which successfully generates completions is as good as any other. Buchner and Wippich (2000) argued that this leads to high levels of response variability (noise) in implicit memory tasks, and they showed empirically that word-stem completion is a statistically less reliable task than recognition. More recently, Ward et al. (2013) demonstrated that the continuous identification (CID) task (described later) also has lower reliability than recognition. The corollary is that tasks with low reliability are unlikely to detect small differences in performance as a function of age. Indeed, Buchner and Wippich demonstrated how differences in measure reliability can explain the age differential pattern of performance on recognition and word-completion tasks. Conversely, Salthouse et al. (1999) reported an instance in which an implicit learning task (serial reaction time) had acceptable reliability levels, and was associated with a small but significant age effect.

There are several other measurement issues that could contribute toward differential age effects on explicit and implicit tasks. Typically the tasks differ in several characteristics, such as the available retrieval cues, the processing demands, and the type of response required, and different tasks are likely to be more or less sensitive to aging depending on their specific requirements and characteristics. For example, it is thought that conceptual processing is affected by age to a greater extent than perceptual processing (Jelicic et al., 1996), and whereas many explicit tasks require conceptual processing (e.g., cued recall), most implicit tasks are perceptual in nature (e.g., word-fragment completion, perceptual identification). Furthermore, there is evidence that production processes tend to diminish with age, while identification processes are relatively spared (e.g., Rybash, 1996), so this may explain why there are larger age effects on explicit tasks such as free recall in comparison to implicit tasks involving simple identification. Another important issue is that indices of explicit and implicit memory have traditionally been captured in separate experimental phases. That is, priming for a subset of previously studied items is usually measured prior to measuring explicit memory for another subset of items (e.g., Mitchell and Bruss, 2003), so it is perhaps not surprising that memory in the latter is weaker, especially in older individuals, due to the longer delay involved (evidence suggests that explicit memory declines rapidly over delays, e.g., Mitchell et al., 1990). Furthermore, even if tasks with comparable characteristics are used, scores may dissociate when they are presented in separate experimental phases because participants may adopt different response strategies or levels of motivation in the two.

From the evidence reviewed above, the following is clear: First, the interpretation that implicit memory is preserved with age is questionable. Second, there are several reasons why outcomes on explicit and implicit memory tests may differ as a function of age, even if the two tap the same underlying memory system. Thus, age-related dissociations between explicit and implicit memory do not necessarily constitute evidence for multiple memory systems. This general viewpoint is not new (see Buchner and Wippich, 2000; Dunn, 2003; Berry et al., 2006, 2008a,b; Nosofsky et al., 2012; Ward et al., 2013), but until recently there have been few attempts to overcome these key measurement issues. This was the main goal in the study by Ward et al. (2013). Healthy young and older adults studied pictures of everyday objects before performing a continuous identification with recognition (CID-R) task. This task captures a measure of recognition and priming for each item concurrently, ensuring that samples of memory are as comparable as possible. In the task, old and new pictures gradually clarified from a background mask and participants were asked to identify the item as quickly as possible (yielding a priming measure) before indicating whether they believed the item had been previously studied (yielding a recognition measure). Obtaining a clear dissociation using this method would constitute more compelling evidence for multiple memory systems relative to when items are judged on two separate occasions (i.e., in separate experimental phases). Two experiments demonstrated significantly weaker recognition memory in older relative to young adults, and there was a clear numerical reduction in priming with age. Importantly, the priming task was found to be objectively less reliable than the recognition task, and the age difference in priming became significant when the data were pooled across experiments to increase power. These findings are consistent with the single-system view.

Some have argued that reduced priming in older compared to young individuals is due to explicit contamination. That is, if participants notice during an implicit test that some items were previously studied (termed test awareness), they may voluntarily attempt to use an explicit processing strategy. For example, in a word-completion task, instead of completing stems or fragments with words that first come to mind, participants may try to explicitly recall items from the prior study phase (discussed in Spaan and Raaijmakers, 2011). Similarly, in a perceptual identification task, participants may try to recall the items shown in the study episode in an attempt to aid their identifications. The use of such a strategy could conceivably lead to an age difference in performance, because it is likely to be more beneficial to young individuals due to their superior explicit memory. The evidence for this argument, however, is mixed. Russo and Parkin (1993) found an age difference in priming on a fragmented picture completion task, but the effect disappeared when available explicit memory was equated between groups by asking young individuals to perform a dual task at study. Geraci and Barnhardt (2010) found greater levels of test awareness and priming in young relative to older adults on word-stem completion and category production tasks, and a greater relationship between the two, which was taken as evidence that test awareness mediates age effects in priming. Moreover, Park and Shaw (1992) demonstrated a small, non-significant age difference in priming on a word-stem completion task, but means were identical for young and older participants who were test unaware (0.08 proportion priming). On the other hand, however, priming effects in other studies have been largely unaffected by test awareness. For example, in Ward et al.'s (2013) study the numerical age difference in priming persisted when the data of aware participants were removed (mean proportions of 0.13 and 0.08 for young and older adults, respectively) (see also Light et al., 2000).

Ward et al. (2013) examined in detail the effect of explicit processing in the CID (continuous identification) task, and found no evidence that this affects the magnitude of priming obtained (Experiments 3A–C). Manipulations were introduced to facilitate successful explicit processing, including informing participants in advance which items were old and new, and varying the ratio of old to new test trials (in order to manipulate the likelihood that participants would become test aware—there is a greater likelihood that participants will become aware when there is a high number of old relative to new test trials). Even when participants received optimal explicit information, priming in young individuals did not differ from that in participants who received no explicit information and did not become test aware (Experiments 3A,B). Moreover, performance was not worsened in young individuals when explicit processing was made disadvantageous by providing incorrect “old/new” test cues (Experiment 3C). This suggests that priming in this task is not affected by optimal or adverse explicit processing, and thus the reliable age difference in priming is unlikely to have been driven by the differential use of an explicit processing strategy by young and older adults. Of course, caution must be taken when interpreting null results. An analysis revealed that Experiment 3A with 107 participants had adequate power to detect a relatively small effect of informing participants, if it had existed (power of 0.82 to detect an effect size of 0.28). The true numerical effect size was tiny (0.03), thus, if real, more than 7000 participants would have been needed to detect the effect statistically. Moreover, in Experiment 3B, the small numerical priming trend was in the opposite direction to that which would be expected if explicit processing were able to boost priming (i.e., greater priming in uninformed relative to informed conditions).

To summarize this section, it appears that both explicit and implicit memory decline with age, although the reduction in priming is usually much smaller than that in recognition, such that high statistical power is needed in order to detect it. Although many studies have reported a non-significant age difference in priming, the power issue combined with evidence that implicit tasks often have low reliability weakens the common claim that implicit memory is completely unaffected by aging. For this reason, these sorts of observations should not be taken as support for the view that explicit and implicit memory are driven by independent memory systems. A range of other studies, including the recent investigation by Ward et al. (2013) which aimed to overcome some critical measurement issues, suggest that there is a general memory decline that is simply picked up more easily on explicit tasks. This supports the view that a single system drives both explicit and implicit memory phenomena.

In the next section we describe how formal computational models have allowed more insight into the problem, and we discuss new evidence in relation to the application of such models to the data of Ward et al. (2013).

## Evidence from formal single and multiple systems models

Computational models can offer considerable theoretical insights into empirical dissociations, as simulations can provide an explicit (in the sense of precise and detailed) account of the cognitive mechanisms underlying task performance. Single-system models have successfully reproduced several dissociations that have previously been taken as support for multiple memory systems (e.g., Nosofsky and Zaki, 1998; Kinder and Shanks, 2001, 2003; Shanks and Perruchet, 2002; Shanks et al., 2003; Berry et al., 2006, 2008a,b, 2010, 2012; Newell and Dunn, 2008; Newell et al., 2010; Dunn et al., 2012; Nosofsky et al., 2012). The Berry et al. single system model assumes that a single memory signal drives performance on explicit and implicit tasks, but that there are in addition independent sources of random noise contributing to performance on these tasks. It assumes that the variance of the noise is typically greater in the implicit task, which is consistent with observations that these tasks have lower reliability than explicit tasks. Ward et al. (2013) previously showed that a larger age difference in recognition than priming could be explained well by this single-system model. A reduction in the strength of the signal gave rise to the pattern of performance seen in aging.

However, rather than simply showing that this single-system model can provide a good fit to the data, a crucial test is to determine whether it provides a better account of the data than multiple-systems models. Here we investigate this by formally comparing the fits of the single-system (SS) model and multiple-systems versions of the model to the data of Ward et al. (2013). To our knowledge this is the first attempt to formally fit multiple-systems models to empirical aging data. We first describe the models before explaining the fitting process and the findings.

In the SS model, it is assumed that every item at test is associated with a memory strength of evidence value, *f*, which is a normally distributed, random variable with mean μ and standard deviation σ_*f*_ (i.e., *f* ~ *N*(μ, σ_*f*_)). Because old items are previously studied, the mean *f* of old items (μ_old_) is greater than that of new items (μ_new_, set to equal zero). In order to derive a recognition judgment for the item, its value of *f* is combined with a value of noise, *e*_r_, which is specific to the recognition task, giving the recognition strength variable *J*_r_:

(1) $$J_{\text{r}}=f+e_{\text{r}}$$

where *e*_r_ is another normally distributed random variable with a mean equal to zero and standard deviation σ_r_ (*e*_r_ ~ *N*(0, σ_r_)). *e*_r_ is uncorrelated with *f*, and represents the influence of non-memorial factors upon task performance (e.g., trial-to-trial variability in the placement of a decision criterion for making an “old” judgment). If *J*_r_ exceeds a threshold, *C*, then the item will be judged old, or else it will be judged new. Thus, when μ_old_ is greater than μ_new_, a greater proportion of old than new items will tend to have values of *J*_r_ that exceed *C*; hence old items will be judged old more often than new ones.

Each item's value of *f* is also used to derive its identification RT (the basis of the priming measure). It is assumed that RT is a decreasing function of *f* such that greater values of *f* will tend to produce shorter identification RTs:

(2) $$\text{RT}=b-sf+e_{\text{p}}$$

where *b* represents the identification RT intercept (and is the expected RT of new items), and *s* represents the rate of change in RT with *f*. Both *s* and *b* serve to scale the identification RT. *e*_p_ is another source of noise, which is also a normally distributed, random variable (*e*_p_ ~ *N*(0, σ_p_)), is uncorrelated with *e*_r_ and *f*, and represents the influence of non-memorial factors upon performance in the identification task (e.g., the influence of extraneous perceptual factors upon identification, such as the amount of perceptual information available from the stimuli when presented at test; see Ostergaard, 1998). Thus, when μ_old_ is greater than μ_new_, old items will tend to have shorter identification RTs than new items (i.e., there will be a priming effect).

Because each item's recognition judgment and identification RT are derived from the same value of *f* in the SS model, it predicts that there will be associations between the recognition judgment and the identification RT. Greater values of *f* will tend to lead to a greater likelihood of an old judgment and also a relatively short identification RT. Thus, within item types, one prediction of the SS model is that there will be fluency effects, in other words that items judged old will tend to have shorter identification RTs than items judged new [i.e., RT(hit) < RT(miss) and RT(false alarm) < RT(correct rejection), where a hit is an old judgment to an old item, a miss is a new judgment to an old item, a false alarm is an old judgment to a new item, and a correct rejection is a new judgment to a new item] (Prediction 1). A second prediction concerns the relative magnitudes of the priming effect across all items and the priming effect for items judged new. The latter priming effect is shown if the identification RTs for old items not recognized (misses) are shorter than the identification RTs for new items judged new (correct rejections), and indicates that a priming effect is occurring even in the absence of overt recognition. In the model, the *J*_r_ values of misses will tend to be larger than those of correct rejections (because misses are old items), but the difference in *J*_r_ between *all* old and new items will tend to be greater than the difference in *J*_r_ to misses and correct rejections because all of the *J*_r_ values for misses and correct rejections are below *C*. Differences in *J*_r_ tend to reflect differences in identification RTs. Thus, the priming effect across all items [i.e., RT(new) − RT(old)] will tend to be greater than the priming effect for items judged new [i.e., RT(correct rejection − RT(miss)] (Prediction 2).

In the multiple-systems 1 (MS1) and multiple-systems 2 (MS2) models (see Berry et al., 2012), recognition judgments and identification RTs are derived in the same manner described above, except that in order to represent the multiple memory systems proposal that the sources of memory driving recognition and priming are distinct, one “explicit” source of memory strength, *f*_r_, is used to derive *J*_r_, and a separate “implicit” source of strength, *f*_p_, is used to derive the RT, where *f*_r_ ~ *N*(μ_r_, σ_*f*_), and *f*_p_ ~ *N*(μ_p_, σ_*f*_). Importantly, μ_r|old_ and μ_p|old_ in the MS1 and MS2 models are free to vary independently of one another. This enables these models to reproduce findings of functional independence, that is, independent variables (e.g., aging) can, in principle, selectively affect the overall strength in one of the memory systems (i.e., selectively affect μ_r|old_ or μ_p|old_).

In the MS1 model, an extra assumption is that the explicit and implicit strengths of a given item are uncorrelated [i.e., *r*(*f*_r_, *f*_p_) = 0], which represents a relatively strong interpretation of the claim that explicit and implicit memory systems are independent. This captures the claim that recognition and priming performance across items is completely uncorrelated (i.e., Tulving et al., 1982). In the MS2 model, the *f*_p_ and *f*_r_ signals of a given item may be correlated, with value *w*. This correlation could arise, for example, as a result of distinctiveness: a particularly distinctive item at study may be encoded strongly into both the explicit and implicit memory systems (and hence both *f*_p_ and *f*_r_ for that item will be relatively high). The MS2 model therefore offers a weaker interpretation of the idea that explicit and implicit memory systems are independent: although independent variables may selectively affect μ_r_ and μ_p_ (i.e., the systems are functionally independent), there may be associations between recognition judgments and identification RTs too. Thus, the MS2 model is more flexible than the SS and MS1 models. In fact, both the SS and MS1 models are special cases of the MS2 model: When *w* = 1 and μ_r_ = μ_p_, the MS2 model is equivalent to the SS model; when *w* = 0, the MS2 model is equivalent to the MS1 model.

## Results: fitting the models to the data of Ward et al. (2013)

**Figure 1:**
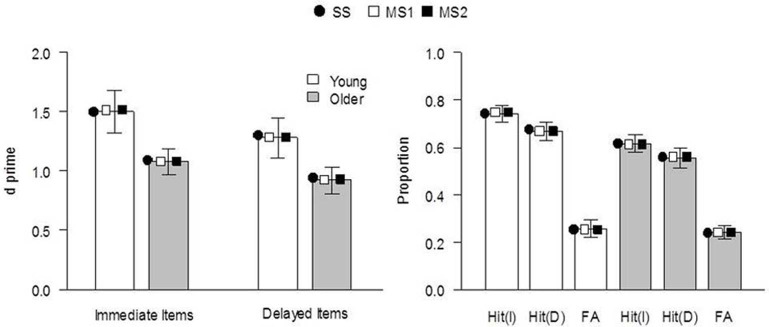
**Recognition performance for immediate and delayed items in Ward et al. (2013), Experiment 1. Left panel:** discriminability performance (*d′*) [significant main effect of age group, *F*_(1, 38)_ = 4.26, *p* = 0.04, η^2^_p_ = 0.10, and delay, *F*_(1, 38)_ = 5.76, *p* = 0.02, η^2^_p_ = 0.13, and no significant interaction, *F*_(1, 38)_ = 0.16, *p* = 0.69]. **Right panel:** proportion of hit and false-alarm responses. Bars indicate experimental data (error bars indicate SE of the mean), and symbols indicate the mean expected result from each model when fit to each individual's data. I, immediate; D, delayed; SS, single-system model; MS1, multiple-systems-1 model; MS2, multiple-systems-2 model.

**Figure 2:**
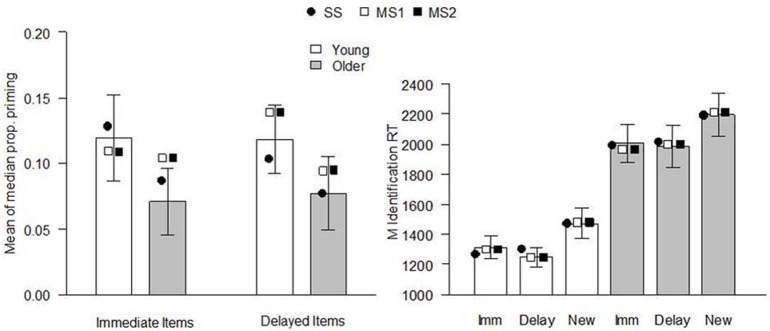
**Continuous identification (CID) task performance in Ward et al. (2013), Experiment 1. Left panel:** priming effects [no significant main effect of age group, *F*_(1, 38)_ = 1.78, *p* = 0.19, or delay, *F*_(1, 38)_ = 0.02, *p* = 0.89, and no significant interaction, *F*_(1, 38)_ = 0.04, *p* = 0.85]. The proportional priming effect was calculated as the difference in the median identification RT to new and old items divided by the median identification RT for new items. Bars indicate the mean of the median priming across participants (error bars indicate SE of the mean), and symbols indicate the mean expected proportional priming (relative to the expected identification RT for new items, see Table 3) from each model when fit to each individual's data. **Right panel:** mean identification RTs (ms) of immediate, delayed, and new items. Bars indicate experimental data (error bars indicate SE of the mean), and symbols indicate the mean expected result from each model when fit to each individual's data. Imm, immediate; Delay, delayed; SS, single-system model; MS1, multiple-systems-1 model; MS2, multiple-systems-2 model.

The models were fit to the data of Ward et al. (2013), Experiment 1. The behavioral procedure and findings were as follows: Healthy young and older adults studied two sets of 30 pictures of everyday objects, separated by a 60 min delay. Immediately following the second study phase, participants performed a CID-R task, which contained the 60 previously studied items (immediate and delayed), as well as an equal number of new items. The delay was included to reduce the strength of memory for a subset of items as much as possible. In the test, old and new pictures gradually clarified from a background mask and participants were asked to identify the item as quickly as possible (priming index) before deciding whether it was presented in either of the earlier study phases (recognition judgment). There were significant main effects of age and delay on recognition (*d*′, see Figure 1), with performance being superior for young relative to older adults at both retention intervals. Recognition was significantly reduced for items studied 60 min prior to testing, relative to those studied immediately before. Priming was calculated for each individual as the difference between the median identification RT for old (immediate and delayed) and new items, in proportion to their baseline (new item) RT [i.e., (RTnew − RTold[immediate or delayed])/RTnew; Figure 2]. Priming was numerically reduced in older compared to young individuals, but there was no significant main effect of age or delay (though as noted above, when Ward et al. pooled data from this and two other similar experiments, the age effect on priming reached significance).

The models were fit to the data using maximum likelihood estimation. An outline is given here; full details of the general fitting procedure can be found in Berry et al. (2012). An automated procedure was used to find the values of the parameters that maximized the summed log likelihood across trials, for each participant. Certain parameter values were fixed (as in Berry et al., 2012): the mean *f* of new items was fixed to zero (i.e., μ_new_ = 0 in the SS model, and μ_r|new_ = μ_p|new_ = 0 in the MS1 and MS2 models), the mean of *e*_p_ and *e*_r_ were also fixed to zero, and the standard deviation of *f* for old and new items was fixed to equal the standard deviation of the noise associated with recognition, that is, $\sigma_{f}=\sigma_{\text{r}}=\sqrt{0.5}$ (and so σ^2^_*J*r_ = σ^2^_*f*_ + σ^2^_r_ = 1). Finally, the value of *s* in the MS1 and MS2 models was fixed to that of the SS model. There are six free parameters in the SS model: μ_immediate_, the mean *f* of the immediate item distribution; μ_delayed_, the mean *f* of the delayed item distribution; *C*, the “old” judgment criterion; *b*, the identification RT intercept; *s* the rate of change in RT with changes in *f*; and σ_p_, the variance of *e*_p_, the noise associated with the priming task. The MS1 model has seven free parameters: μ_r|immediate_, the mean explicit memory strength of the immediate item distribution; μ_p|immediate_, the mean implicit memory strength of the immediate item distribution; μ_r|delayed_, the mean explicit memory strength of the delayed item distribution; μ_p|delayed_, the mean implicit memory strength of the immediate item distribution; *C*, the “old” judgment criterion; *b*, the identification RT intercept; and σ_p_, the variance of *e*_p_. The MS2 model has eight free parameters: like the MS1 model, μ_r|immediate_, μ_p|immediate_, μ_r|delayed_, μ_p|delayed_, *b*, *C*, and σ_p_ are free, but additionally, *w*, the correlation between *f*_r_ and *f*_p_, is a free parameter.

The likelihood of each identification RT (RT) and recognition judgment *Z* on a given trial is given as follows:

(3) $$\begin{array}{l} L\left(Z,RT|I\right)=\left[\Phi \left(C_{j}|\mu_{J\text{r}|\text{RT},I},\sigma_{J\text{r}|\text{RT}}^{2}\right)\text{​}-\text{​}\Phi \left(C_{j-1}|\mu_{J\text{r}|\text{RT},I},\;\sigma_{J\text{r}|\text{RT}}^{2}\right)\text{​}\right]\\ \;\times \;\phi (RT|b-s\mu_{\text{p}|I},\;\sigma_{\text{RT}}^{2}) \end{array}$$

where *I* is the item type and *I* = new, immediate, delayed; Φ is the cumulative normal distribution function; ϕ is the normal density function; and σ^2^_RT_ = *s*^2^σ^2^_*f*_ + σ^2^_p_. *j* = 1 when *Z* = “new,” and *j* = 2 when *Z* = “old”; *C*_0_ = −∞, *C*_1_ = *C* and *C*_2_ = ∞. μ_*J*r|RT,*I*_ and σ^2^_*J*r|RT_ are the mean and variance of the conditional distribution of *J*_r_ given RT, calculated as

(4) $$\mu_{J\text{r}|\text{RT},I}=\mu_{\text{r}|I}-\frac{ws\sigma_{f}^{2}\left(\text{RT}-b+s\mu_{\text{p}|I}\right)}{s^{2}\sigma_{f}^{2}+\sigma_{\text{p}}^{2}}$$

and

(5) $$\sigma_{J\text{r}|\text{RT}}^{2}=\sigma_{f}^{2}+\sigma_{\text{r}}^{2}-\frac{w^{2}s^{2}\sigma_{f}^{4}}{s^{2}\sigma_{f}^{2}+\sigma_{\text{p}}^{2}}$$

where μ_r|new_ = 0 when *I* = new, μ_r|immediate_ ≥ 0 when *I* = immediate, and μ_r|delayed_ ≥ 0 when *I* = delayed; μ_p|new_ = 0 when *I* = new, μ_p|immediate_ ≥ 0 when *I* = immediate, and μ_p|delayed_ ≥ 0 when *I* = delayed. In the SS model, μ_r|new_ = μ_p|new_ = 0, μ_r|immediate_ = μ_p|immediate_, μ_r|delayed_ = μ_p|delayed_, and *w* = 1. In the MS1 model *w* = 0. In the MS2 model 0 ≤ w ≤ 1.

Given the best fitting parameter values for a model, the expected model results can be calculated analytically (see Table 1).

**Table 1:** **Expected values for measures of recognition and priming in the models**.

**Measure**	**Model expected value**
*P*(Hit|immediate)	1—Φ (*C*—μ_r|immediate_)
*P*(Hit|delayed)	1—Φ (*C*—μ_r|delayed_)
*P*(False Alarm)	1—Φ (*C*)
*d*'_Immediate_	μ_r|immediate_
*d*'_Delayed_	μ_r|delayed_
*E*[RT|new]	*b*
*E*[RT|immediate]	*b*—*s*μ_p|immediate_
*E*[RT|delayed]	*b*—*s*μ_p|delayed_
Proportional priming measure (immediate items)	*s*μ_p|immediate_/*b*
Proportional priming measure (delayed items)	*s*μ_p|delayed_/*b*

In the SS, MS1, and MS2 models, the expected values of RT conditional on judgment *Z* are given by the following:

(6) $$E\left[\text{RT}|Z,I\right]=b-s\mu_{\text{p}|I}+\frac{sw\sigma_{f}^{2}}{\sigma_{J\text{r}}}\frac{\phi \left(\frac{C_{j}-\mu_{\text{r}|I}}{\sigma_{J\text{r}}}\right)-\phi \left(\frac{C_{j-1}-\mu_{\text{r}|I}}{\sigma_{J\text{r}}}\right)}{\Phi \left(\frac{C_{j}-\mu_{\text{r}|I}}{\sigma_{J\text{r}}}\right)-\Phi \left(\frac{C_{j-1}-\mu_{\text{r}|I}}{\sigma_{J\text{r}}}\right)}$$

where $\sigma_{J\text{r}}=\sqrt{\sigma_{f}^{2}+\sigma_{\text{r}}^{2}}$. *j* = 1 when *Z* = N, and *j* = 2 when *Z* = O; *C*_0_ = −∞, *C*_1_ = *C* and *C*_2_ = ∞.

Thus, Equation 6 gives the expected RT of hits for immediate items when *I* = immediate and *Z* = O; it gives the expected RT of hits for delayed items when *I* = delayed and *Z* = O; it gives the expected RT of false alarms when *I* = new and *Z* = O. Similarly, Equation 6 gives the expected RT of misses for immediate items when *I* = immediate and *Z* = N; it gives the expected RT of misses for delayed items when *I* = delayed and *Z* = N; and gives the expected RT of correct rejections when *I* = new and *Z* = N.

**Figure 3:**
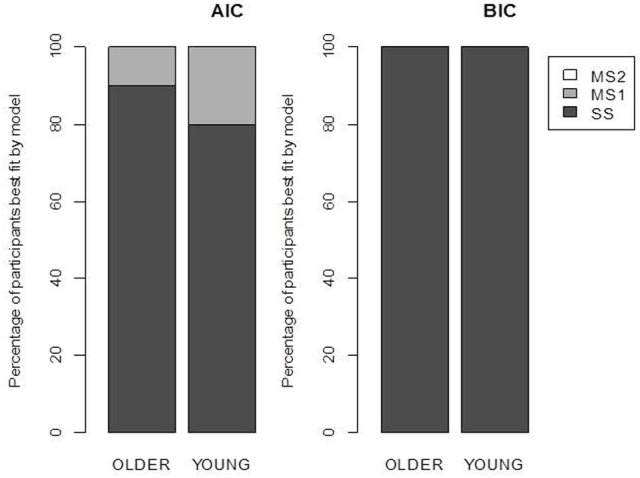
**Model selection results**. Each bar represents the percentage of participants best fit by each model according to the Akaike Information Criterion (AIC) and the Bayesian Information Criterion (BIC). SS, single-system model; MS1, multiple-systems-1 model; MS2, multiple-systems-2 model.

The maximum likelihood estimates of the parameters are given in Table 2, and the goodness of fit of the models is given in Table 3. Unsurprisingly, the greater flexibility of the MS2 model allows it to provide the best fit to the data, as indicated by the smallest ln(*L*) values. However, the Akaike Information Criterion (AIC, Akaike, 1973) and Bayesian Information Criterion (BIC, Schwarz, 1978) model evidence measures, which penalize models with more free parameters and which in the case of the BIC also take into account the number of data points being fit, clearly favored the SS model over the MS1 and MS2 models, as indicated by the lower AIC and BIC values (Burnham and Anderson, 1998). Figure 3 shows the proportion of participants that were best fit by each of the models. It is clear that the SS model was the best fitting model by the AIC and BIC measures for the vast majority of participants; a small minority were best fit by the MS1 model. None were best fit by the MS2 model.

**Table 2:** **Mean and standard deviation (in parentheses) of the parameter estimates of the models (derived by maximum likelihood estimation)**.

**Parameter**	**SS**		**MS1**		**MS2**	
	**Young**	**Older**	**Young**	**Older**	**Young**	**Older**
μ_r|immediate_	1.50 (0.76)	1.10 (0.45)	1.52 (0.75)	1.08 (0.48)	1.52 (0.75)	1.09 (0.48)
μ_r|delayed_	1.31 (0.73)	0.95 (0.50)	1.29 (0.74)	0.93 (0.50)	1.29 (0.74)	0.93 (0.50)
μ_p|immediate_	= μ_r|immediate_	= μ_r|immediate_	1.23 (1.05)	1.16 (0.62)	1.22 (1.22)	1.14 (0.65)
μ_p|delayed_	= μ_r|delayed_	= μ_r|delayed_	1.96 (1.42)	1.34 (0.76)	1.96 (1.96)	1.41 (0.72)
*w*	= 1	= 1	= 0.00	= 0.00	0.56 (0.42)	0.56 (0.41)
*C*	0.79 (0.64)	0.77 (0.43)	0.79 (0.64)	0.76 (0.43)	0.79 (0.64)	0.76 (0.43)
*b*	1470 (442)	2189 (635)	1480 (461)	2210 (636)	1480 (461)	2210 (636)
*s*	140 (100)	199 (166)	= SS	= SS	= SS	= SS
σ_p_	630 (197)	936 (315)	626 (193)	932 (313)	625 (193)	931 (312)

A value preceded by an equal sign indicates that the value was fixed. The parameters σ_f_ and σ_r_ are not shown, but in all models their value was fixed to σ_f_ = σ_r_ = $1/\sqrt{2}$.

**Table 3:** **Goodness of fit of the models**.

	**SS**				**MS1**				**MS2**				
	***p***	**ln(*L*)**	**AIC**	**BIC**	***p***	**ln(*L*)**	**AIC**	**BIC**	***p***	**ln(*L*)**	**AIC**	**BIC**
Young (*n* = 20)	6	−19332.1	**38904.2**	**39593.9**	7	−19330.4	38940.7	39745.3	8	−19317.1	38954.2	39873.7
Older (*n* = 20)	6	−18612.9	**37465.7**	**38144.6**	7	−18611.1	37502.1	38294.2	8	−18604.2	37528.4	38433.6

AIC stands for the Akaike (1973) Information Criterion, calculated as AIC = −2ln(L) + 2P, where P = p × n is the total number of free parameters for each fit, where p is the number of free parameters for each model, and n is the number of participants modeled in each experiment; BIC stands for the Bayesian Information Criterion (Schwartz, 1978), calculated as BIC = −2ln(L) + Pln(q), where q is the number of data points fit: q Young = 2315, q Older = 2117. Bold indicates best fitting model by the AIC or BIC.

**Figure 4:**
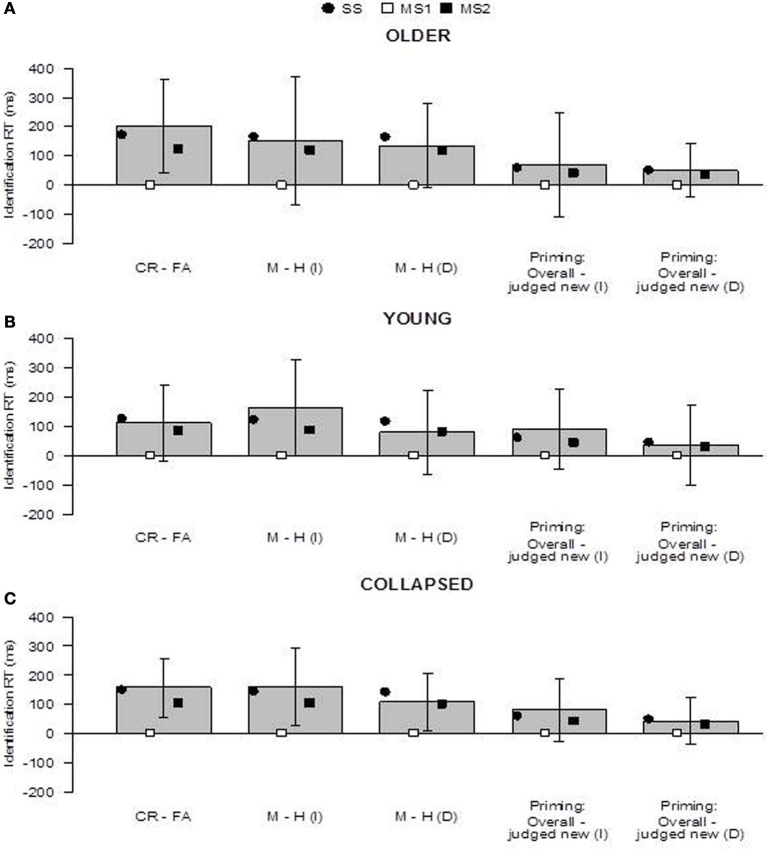
**Predictions of the SS, MS1, and MS2 models (Berry et al., 2012) in the older group (panel A), younger group (panel B), and collapsed across groups (panel C)**. “Priming: Overall - judged new” refers to the difference in the overall priming effect and the priming effect for items judged new. Prediction 1 concerns whether fluency effects occur within new and old items (i.e., CR − FA and M − H). Prediction 2 concerns whether the magnitude of the priming effect overall (across all items) is greater than the priming effect for items judged new. Bars indicate 95% confidence intervals. I, immediate; D, delayed; CR, correct rejection, FA, false alarm; M, miss; H, hit; SS, single-system model; MS1, multiple-systems-1 model; MS2, multiple-systems-2 model.

The expected model results are presented in Figures 1, 2, 4. All models reproduced the trend for recognition memory to be greater for immediate than for delayed items. These effects all arise because μ_r_ is larger for young than for old participants and for immediate vs. delayed items, as shown in the top 2 rows of Table 2. The models all also predicted that priming in the young adult group would be greater than that in the older adult group (Figures 1, 2). With regards to the expected proportional priming effects (Figure 2; calculated as specified in Table 1), the SS model results mirror those of the recognition data: that is, priming in the older group is lower than that of the young group, and priming for immediate items is greater than that of delayed items. Under the MS1 and MS2 models, priming was lower in the older group than the young group for both immediate and delayed items. Furthermore, in the older group, the expected priming effect for delayed items was less than for immediate items; however, in the young group, the expected priming for delayed items was greater than for immediate items.

The results concerning the specific predictions of the models are shown in Figure 4. In both groups, in line with the advance predictions of the SS model, there were trends for items judged old to have shorter identification RTs than items judged new (i.e., fluency effects, Prediction 1). This was the case within new items [i.e., RT(FA) < RT(CR)] and also within immediate and delayed old items [i.e., RT(H) < RT(M) for both item types], but this was only reliable for new items in the older group, *t*_(19)_ = 2.61, *p* = 0.02. Similarly, in both groups, and for both immediate and delayed items, there was a trend for the priming effect to be greater than the priming effect for items judged new (Prediction 2; but all differences were non-significant). When the data were collapsed across groups, however, the fluency effects within new, immediate, and delayed items were reliable (Figure 4C): new items, *t*_(39)_ = 3.14, *p* = 0.003; immediate items, *t*_(39)_ = 2.42, *p* = 0.02; delayed items, *t*_(39)_ = 2.21, *p* = 0.03. These differences are as predicted by the SS model, but not the MS1 model; they were reproduced by the MS2 model. The differences in the magnitude of priming overall and for items judged new were not reliable within the collapsed data: immediate items, *t*_(39)_ = 1.50, *p* = 0.14; delayed items, *t*_(39)_ = 1.09, *p* = 0.28.

In sum, the patterns of recognition and priming for young and older adults reported in Ward et al. (2013) are largely consistent with the single-system model, according to which any differences in performance with age are primarily consequences of a reduction in the strength of a single underlying memory signal. Two plausible multiple-system models fared considerably worse when using advanced model selection techniques. In the best fits of the MS1 and MS2 models, the mean strength of the implicit memory signal μ_p_ was lower for the older group than the young group (Table 2). From a multiple systems perspective, this could suggest that aging affects both the explicit and implicit memory systems, yet the poor performance of the MS1 model is evidence against the idea that the memory signals driving recognition and priming are completely independent. The qualitative results of the more flexible MS2 model were similar to the SS model. According to this model, there is a substantial degree of correlation between the explicit and implicit memory strengths of an individual item. However, the SS model was resoundingly preferred by the AIC and BIC measures.

## Conclusions

The evidence presented in this article strongly draws into question the claim that implicit memory is preserved in normal aging. When appropriate methods and controls are used, priming is reduced in older individuals compared to young, although the effect is smaller than the reduction in explicit memory. Indeed, age differences in priming rarely reach significance, but this is likely to be due to a combination of low statistical power and low measure reliability. The profile of memory decline in aging suggests that there is a general impairment to a unitary memory system. Consistent with this, we provide new evidence that the patterns are more compatible with a formal single-system model than feasible multiple-systems alternatives.

It is important to note that even though age effects on priming may be small and hard to detect in low-powered studies, the single-system model emphatically predicts that such effects exist. This can be seen in the left panel of Figure 2, where the predictions of the model are shown for the Ward et al. (2013) data. Indeed, the predictions of the SS model are strongly constrained: whenever it predicts an age effect on recognition (reflected in a lower value of μ_r_ for older relative to young participants, see Table 2), it also predicts an effect on priming. It makes this prediction simply because recognition and priming are based on the same underlying signal and μ_r_ = μ_p_. In one sense, therefore, it is important that studies are conducted which have adequate power to detect an effect of age on priming: the SS model could be falsified by a high-powered study which failed to detect such an effect. But in another sense it is less important whether age effects on priming are observed or not. All the models can predict such an effect (see Figure 2), and a far more discriminating test is to determine via formal model-fitting techniques (computing AIC and BIC, for instance) which model provides the best fit to the data. Put differently, little will be gained in our view from further studies asking whether age effects on priming reach the rather arbitrary cliff-edge criterion of *p* < 0.05. Instead, more will be learned from taking the underlying quantitative data and asking which theoretical model provides the best fit.

The results are consistent with previous applications of the single-system model to data from individuals with amnesia. For example, Conroy et al. (2005) reported intact priming on a word CID task in individuals with amnesia due to damage to the hippocampus and medial temporal lobe despite substantial impairment to recognition memory. This type of dissociation has been taken as strong evidence for multiple-systems views. The SS model was able to closely fit this pattern (Berry et al., 2012) but, like the present study, the model predicted a small deficit in priming in amnesia. Although controversial, this is consistent with proposals that, when carefully examined, priming is not completely preserved in amnesia (e.g., Ostergaard, 1999). Another commonly cited strand of evidence for multiple systems is that manipulations tend to produce different effects on tests of recognition and priming in healthy young individuals. For example, Butler and Klein (2009) showed that manipulating attention at study resulted in chance-level recognition for ignored items, whereas priming for such items was robust (see also Vuilleumier et al., 2005). The study by Berry et al. (2010) did not replicate this pattern: when recognition was at chance, priming was also absent. Moreover, the data patterns were again consistent with the predictions of the single-system model.

Lastly, the multiple-systems account argues that an implicit memory system is resistant to age-related decline, but in order to make this claim in the future one must provide evidence of completely equivalent priming in young and older individuals, or demonstrate conditions in which priming is greater in older relative to young adults, while at the same time explicit memory is weaker. This kind of double dissociation would present a challenge to the single system model, as the pattern would not occur if performance on explicit and implicit tasks is driven by a single memory signal. We hope that this discussion will lead to further useful developments in this longstanding debate.

### Conflict of interest statement

The authors declare that the research was conducted in the absence of any commercial or financial relationships that could be construed as a potential conflict of interest.
